# Anti-Inflammatory Neutrophil Functions in the Resolution of Inflammation and Tissue Repair

**DOI:** 10.3390/cells11244076

**Published:** 2022-12-16

**Authors:** Waywen Loh, Sonja Vermeren

**Affiliations:** Centre for Inflammation Research, Institute for Regeneration and Repair, The University of Edinburgh, Edinburgh EH10 5HF, UK

**Keywords:** neutrophil, macrophage, inflammation, apoptosis, neutrophil extracellular trap, efferocytosis, resolution, repair

## Abstract

Neutrophils are highly abundant circulating leukocytes that are amongst the first cells to be recruited to sites of infection or sterile injury. Their ability to generate and release powerful cytotoxic products ties with their role in host defence from bacterial and fungal infections. Neutrophilic inflammation is tightly regulated to limit the amount of ‘bystander injury’ caused. Neutrophils were in the past regarded as short-lived, indiscriminate killers of invading microorganisms. However, this view has changed quite dramatically in recent years. Amongst other insights, neutrophils are now recognised to also have important anti-inflammatory functions that are critical for the resolution of inflammation and return to homeostasis. This minireview focusses on anti-inflammatory neutrophil functions, placing a particular focus on recent findings linked to neutrophil cell death, several types of which may be anti-inflammatory (apoptosis, secondary necrosis, and neutrophil extracellular traps). These are discussed together with features that may further promote the clearance of dead cells by efferocytosis and reprogramming of macrophages to promote resolution and repair.

## 1. Introduction

The terminally differentiated neutrophils represent a first cellular response to bacterial and fungal infections. Upon activation, neutrophils adhere to the vessel wall, undergo transendothelial migration and migrate to sites of infection or sterile injury by following gradients of chemokines and chemoattractants. Neutrophils kill germs by making use of specialised effector functions, phagocytosis, degranulation, release of neutrophil extracellular traps (NETs), employing an arsenal of cytocidal compounds, including, reactive oxygen species (ROS), powerful proteases, and antimicrobial peptides. These weapons are released intracellularly following the uptake of microbes, or employed extracellularly to kill microorganisms [1]. Due to the large number and short lifespan of neutrophils (see also below) and the indiscriminate action of neutrophilic cytocidal compounds, neutrophils were long thought of as transcriptionally inactive and rather primitive killers that are capable of inflicting severe bystander injury. With the advent of improved techniques, including transcriptome analysis and powerful intravital imaging, this view has been revised in recent years. Neutrophils are increasingly recognised as a highly plastic, heterogenous population of transcriptionally active cells that hold diverse functions in health and disease [2,3,4,5,6,7,8]. Beyond their well-established function in host defence, neutrophils actively participate in modulating, and indeed orchestrating, the adaptive immune response. As part of this, they hold important anti-inflammatory functions in addition to their much better characterised pro-inflammatory functions [9,10]. This is perhaps most obvious in the context of sterile injury, which, like infections, causes neutrophil recruitment. Danger-associated molecular patters (DAMPs) are released by dying host cells, initiating the generation of neutrophil-active chemokines by tissue resident sentinels. Formylated mitochondrial peptides, which bind formylated peptide receptors FPR1/2, are powerful neutrophil chemoattractants in their own right [11]. The observation that FPR1/2 deficient mice, in which neutrophil recruitment to sterile wounds is reduced, are characterised by delayed wound healing [12] suggests the existence of anti-inflammatory neutrophil functions in sterile wounds. Neutrophils may in such situations promote tissue repair by clearing necrotic tissue and debris by phagocytosis, preventing the induction of persistent inflammation by necrotic cells. Clearing of damaged tissue was observed in the liver in response to burn injury. Interestingly, despite a wealth of evidence supporting neutrophils promoting liver inflammation [13], depletion of neutrophils in this acute model of liver injury, as well as in a range of experimental liver inflammation/fibrosis models delayed repair [14,15,16,17]. Similarly, in some other situations, e.g., peripheral nerve damage, neutrophil depletion again delayed the clearance of debris [18], suggesting that such anti-inflammatory neutrophil functions may not be unusual.

This review aims to provide a concise update on neutrophil anti-inflammatory functions. In particular, we focus on the notion of anti-inflammatory functions of apoptotic, necrotic, and viable neutrophils, as well as those associated with NETs.

## 2. Neutrophils Are Short-Lived

In humans, 1–2 × 10^11^ neutrophils are generated every day by haematopoiesis in the bone marrow [19]. Once fully matured, these neutrophils are released into the circulation in a circadian fashion in human and mouse [20,21]. Neutrophil trafficking is dependent upon chemokines and their cognate receptors. In the mouse, CXCL12 (which binds CXCR4) mediates retention in the bone marrow of nascent cells, while CXCL1/2 (which bind CXCR2) mediate neutrophil release into the circulation. Over the course of the day, neutrophils age, with both ageing and lifespan regulated in a gut microbiota-dependent fashion [22,23]. This affects receptor expression, rendering aged neutrophils more responsive to CXCR4 signalling [24,25]. Although the lifespan of activated neutrophils can be significantly extended [26], aged circulating neutrophils are programmed to home to the bone marrow to undergo apoptosis and be cleared by efferocytosis by resident macrophages (see below for details). Outside of the bone marrow, removal of dead neutrophils by macrophages also occurs in the spleen, and in a Kupffer cell-dependent fashion in the liver [27].

In homeostasis, circulating neutrophil counts are constant due to the balanced production of new neutrophils by granulopoiesis and elimination of aged cells at ~1 day. In acute inflammation or infection, the titre of circulating neutrophils is increased due to an increase in the lifespan of circulating neutrophils that is driven by the life-extending effect of pro-inflammatory cytokines (e.g., GM-CSF, G-CSF, IL-8) [28,29] and hypoxia [30,31]. In contrast, in severe systemic bacterial infection, neutrophil turnover is increased due to the need to kill invading bacteria, which prompts increased neutrophil death (see below), promoting emergency haematopoiesis for increased myeloid cell production. This results in the release of immature neutrophils (band cells) from the bone marrow [32].

## 3. Neutrophil Cell Death Pathways

**Figure 1:**
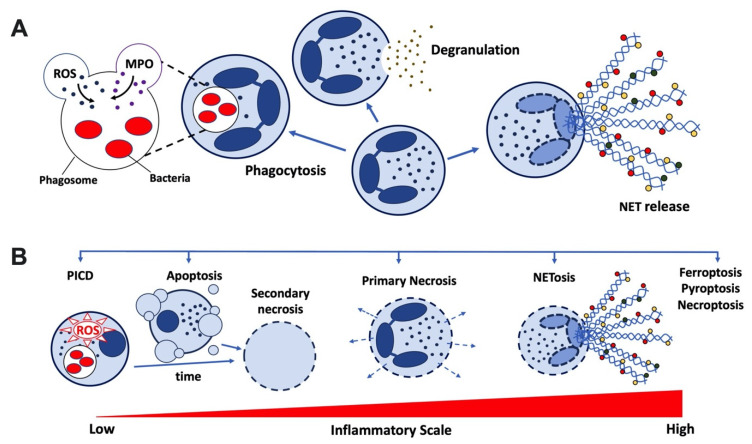
**Overview of neutrophil functions and death**. Neutrophils are derived by haematopoiesis in the bone marrow, and released into the circulation. (**A**) Effector functions important for host defence, and relevant to the induction of neutrophil death include phagocytosis, degranulation, and NET release. Additional neutrophil functions exist but are not included here. (**B**) Unless activated and recruited to tissues, after circulating for ~1 day, aged neutrophils home to sites of haematopoiesis and undergo constitutive apoptosis. Phagocytosis induced cell death (PICD), or immune complex induced apoptosis, are dependent upon ROS production. Apoptotic cells display ‘eat-me’ signals, e.g., PS, are characterised by an intact plasma membrane and condensed chromatin. Note, apoptotic blebs/extracellular vesicles are not included into neutrophils undergoing PICD here for clarity’s sake. Unless cleared in a timely fashion, apoptotic neutrophils loose membrane integrity and undergo secondary necrosis. Due to the apoptotic machinery, secondary necrotic neutrophils are characterised by degradation of intracellular proteins and chromatin, rendering their contents less inflammatory than those of cells that have undergone primary necrosis. Adverse environmental conditions may result in sudden primary necrosis. Plasma membrane integrity is compromised, causing release of danger associated molecular patterns (DAMP) into the extracellular milieu. NET release is associated with a pro-inflammatory form of cell death, NETosis, which involves exteriorisation of decondensed chromatin decorated with cytotoxic proteins, including histones, neutrophil elastase, myeloperoxidase, as well as antibacterial peptides, indicated here by coloured circles. Other highly pro-inflammatory cell deaths include ferroptosis, which occurs as the result of excessive accumulation of intracellular iron; pyroptosis as the result of caspase 1 activation; and necroptosis, a programmed type of necrosis.

Cell death mechanisms that apply to neutrophils include apoptosis, necrosis, ferroptosis, necroptosis (also known as regulated necrosis), pyroptosis, as well as NETosis (Figure 1). The molecular mechanisms of these different types of cell death have been reviewed in detail elsewhere [33,34,35] and are not covered here. Rather, we focus on anti-inflammatory functions of neutrophils undergoing some of these cellular deaths.

In general, neutrophil apoptosis, where the integrity of the plasma membrane is preserved, maintains homeostasis, or elicits anti-inflammatory and pro-resolution responses, respectively. This contrasts with lytic forms of cell death (necrosis, necroptosis, pyroptosis, ferroptosis, and NETosis) that lead to the rupture of the plasma membrane. Harmful intracellular contents are released, and therefore these types of death are regarded as largely pro-inflammatory.

## 4. Neutrophil Apoptosis—A Powerful Anti-Inflammatory Signal

Neutrophil apoptosis is triggered in aged neutrophils as part of homeostasis, but it may also be induced in specific situations. For example, phagocytosis and intracellular killing of serum or antibody-opsonised pathogens induces a specialised cellular differentiation programme that culminates in phagocytosis-induced cell death (PICD) ([36,37,38,39] and see ROS below). Given the anti-inflammatory nature of neutrophil apoptosis, this mechanism is perfectly suited for promoting tissue repair once the infection has been cleared.

In the absence of pathogens, insoluble antibody complexes, powerful pro-inflammatory stimuli that are abundant in biological fluids in autoimmune disease (e.g., the synovial fluid in rheumatoid arthritis), are internalised by neutrophils by receptor-dependent macropinocytosis [40]. Using a separate pathway, the immune complexes also promote ROS-dependent neutrophil apoptosis. Immune complex-induced neutrophil apoptosis is mechanistically distinct from PICD [40,41,42]. This anti-inflammatory mechanism is likely relevant for immune complex-driven autoimmune diseases, such as rheumatoid arthritis and lupus [43]. In addition, it may be involved in clearing excess antibody and promoting repair processes after infectious diseases, when rheumatoid factor, an Fc region binding IgM, is temporarily expressed, generating large circulating immune complexes [44,45].

### 4.1. Apoptotic Neutrophils Generate ‘Find-Me’ and ‘Eat-Me’ Signals to Promote Efferocytosis and Macrophage Polarisation

**Figure 2:**
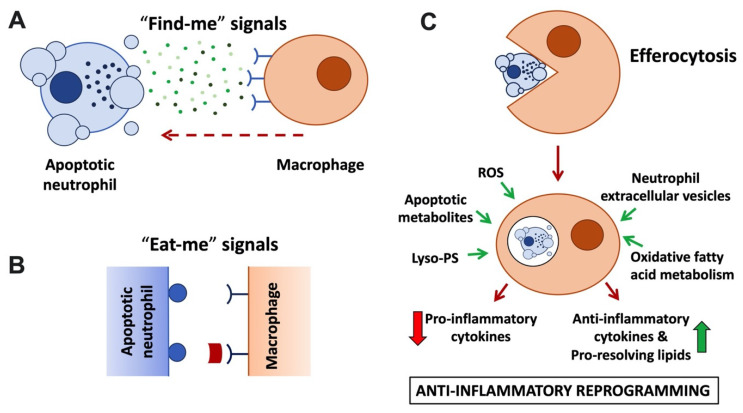
**Anti-inflammatory neutrophil functions linked to apoptosis and efferocytosis.** (**A**) Apoptotic neutrophils and their extracellular vesicles release ‘find-me’ signals, including nucleotides (ATP, UTP), lysophosphatidylcholine, and sphingosine-1-phosphate to attract macrophages. ‘Find-me’ signals bind to cognate receptors on macrophages. (**B**) Apoptotic neutrophils and blebs display ‘eat-me’ signals on their plasma membrane. These include phosphatidylserine (PS) and lyso-PS, which are recognised by macrophage receptors (e.g., BAI1 and TIM family) or that are recognised via bridging molecules, such as protein S and Gas6 that bind to TAM family macrophage receptors. (**C**) Neutrophil efferocytosis. Macrophages engulf apoptotic neutrophils and digest them in an immunologically silent fashion in an efferosome or LAPosome. Released apoptotic cell and fatty acid metabolites ligate metabolite sensing receptors (LXR, PPAR), upregulating transcription of anti-inflammatory cytokines and potentiating efferocytosis. Other signals promoting macrophage reprogramming to the anti-inflammatory phenotype (also known as M2) include ROS, lyso-PS, and neutrophil extracellular vesicles.

Irrespective of its mode of induction, by maintaining the integrity of its plasma membrane, neutrophil apoptosis offers a window of opportunity for clearance of the dead cells without eliciting inflammation. To this end, apoptotic neutrophils promote their own clearance by macrophages via a specialised phagocytosis mechanism known as efferocytosis (Figure 2; reviewed in [46,47]). Efferocytosis not only prevents secondary necrosis of the apoptotic neutrophils; it is also anti-inflammatory, promotes pro-resolving pathways, and initiates repair.

### 4.2. ‘Find-Me’ Signals

Recognition of apoptotic cells by macrophages is promoted via soluble mediators, or ‘find-me’ signals, that are actively released by apoptotic cells and/or extracellular vesicles (Figure 2A; reviewed by [48]). Low concentrations of nucleotides ATP and UTP released by apoptotic neutrophils act as ‘find-me’ signals that bind to the macrophage P2Y2 purinergic receptor [49], while the lipid mediators lysophosphatidylcholine (LPC) and sphingosine 1-phosphate bind to macrophage G2A and S1P1-5 receptors, respectively [50,51]. Besides acting as chemoattractants, ‘find-me’ signals were also reported to have immunomodulatory functions, enhancing the phagocytic activity of macrophages, and mediating anti-inflammatory effects by modulating cytokine production [52,53,54], although some of the underlying mechanisms remain to be fully explored. A recent metabolomics analysis performed with a range of apoptotic immune cells suggested that shared core metabolites act in a cooperative fashion as ‘find-me’ signals [55].

### 4.3. ‘Eat-Me’ Signals

Apoptotic cells also display prominent ‘eat-me’ signals on their surface. These ‘eat-me’ signals are recognised by specialised phagocytic receptors (Figure 2B) and trigger internalisation of the apoptotic corpse. The most prominent ‘eat-me’ signal is the display of the membrane lipid phosphatidylserine (PS) on the outside of the cell. Viable cells are characterised by phospholipid asymmetry. This is due to flippase transporters, which actively limit PS to the inner leaflet of the plasma membrane. In apoptotic cells, flippases are inactivated in a caspase-dependent fashion; simultaneously, scramblases are activated, further disturbing PS asymmetry [56,57,58]. Recognition of PS can be direct via PS receptors, including BAI1 or TIM (T cell/transmembrane, immunoglobulin, and mucin) family receptors. In addition, Tyro/Axl/Mer (TAM) family receptors recognise PS indirectly via soluble bridging molecules, such as protein S and Gas6 (reviewed by [59]). Interestingly, in addition to enabling efferocytosis, TAM receptor activation was reported to elicit additional anti-inflammatory functions, suppressing inflammatory cytokine production and promoting macrophage polarisation toward an alternative or M2-like phenotype [60,61]. A number of ‘eat-me’ signals other than PS exist, e.g., calreticulin and pentraxin 3; these may be expressed in a cell type-specific fashion and act to further promote efferocytosis alongside PS.

In contrast with apoptotic neutrophils, viable cells display ‘do not eat-me signals’ on their surface which interfere with their uptake by macrophages by efferocytosis. ‘Do not eat-me signals’ even counteract the internalisation of PS-displaying cells [62]. For example, the ‘do not eat-me’ signal CD47 binds to its macrophage receptor SIRPα, interfering with the internalisation of bound cells by employing the phosphatase SHP1/2 (for details see review [63]).

### 4.4. Efferocytosis

Following uptake of the apoptotic neutrophil by the macrophage by efferocytosis, degradation of the apoptotic cell ensues (Figure 2C). Degradation was reported to occur by a Rab5, Rab7, and Rab17-dependent variant of the traditional endocytic pathway in an immunologically silent fashion [64,65]. Separately, efferocytosis was also reported to occur via an LC3-associated phagocytosis pathway, short LAP, a non-canonical form of autophagy [66]. LAP stands in sharp contrast to the pro-inflammatory degradative pathway by which degradation of microbes occurs following phagocytosis. LAP employs the phagocyte’s autophagic machinery to degrade the internalised apoptotic material in an anti-inflammatory fashion. Instead of generating an autophagosome, in LAP, LC3 is conjugated to the phagosome or ‘LAPosome’ [66,67]. LAP-deficient animals accumulate apoptotic bodies in their tissues that are reminiscent of the dead cells found in the circulation of systemic lupus erythematosus (SLE) patients, and genetic links between autoimmunity and defective LAP have been identified [68,69]. As with classical autophagy, LAP ultimately results in the release of apoptotic metabolites into the macrophage; it upregulates fatty acid oxidation, which has been proposed to be critical for the anti-inflammatory nature of efferocytosis.

### 4.5. Neutrophil-Dependent Macrophage Reprogramming

Efferocytosis has long been regarded as non-inflammatory. This is (i) because it avoids the release of neutrophil granule contents, limiting tissue injury [70], and (ii) because uptake of apoptotic neutrophils by macrophages was observed to induce their production of anti-inflammatory cytokines (e.g., TGF-β and IL-10) and pro-resolving lipid species (resolvins D1, D2, E4, lipoxin A4 and maresin) at the expense of pro-inflammatory cytokines (e.g., TNF, IL-1β and IL-12) (Figure 2C; [71,72,73]). This stands in sharp contrast to the pro-inflammatory cytokine profile observed after phagocytosis of microbes and nurtured the notion that efferocytosis promotes macrophage polarisation towards a pro-resolution phenotype (also referred to as M2). While the mechanism underpinning the switch from pro- to anti-inflammatory cytokines has not yet been elucidated in its entirety, recent studies have suggested an involvement of metabolic modulation of macrophages post-efferocytosis in conjunction with metabolite sensing nuclear receptors to fine-tune anti-inflammatory processes and regulate the transcription of pro- and anti-inflammatory cytokines. Apoptotic cell-derived metabolites were shown to drive enhanced transcription of engulfment-related genes, including PS receptors and bridging molecules, further increasing macrophage efferocytosis capacity. In addition, genetic experiments identified roles of nuclear fatty acid liver X receptors (LXR) and peroxisome-proliferator-activated receptors (PPAR) for upregulation of TGF-β and IL-10, and the suppression of TNF, IL-1β, and IL-12 [74,75,76]. Recent elegant studies identified that oxidative fatty acid metabolism specifically promotes IL-10 production by macrophages [11]. Additionally, amino acids obtained from apoptotic cells, such as arginine, are metabolised promoting the long-term potentiation of efferocytosis and contributing to the resolution of inflammation [77].

### 4.6. Neutrophil Extracellular Vesicles

In recent years, extracellular vesicles (EVs) have emerged as a means by which cells communicate with one another in a range of situations. Neutrophilic EVs (sometimes also referred to as microvesicles or ectosomes) are produced by membrane blebbing (Figure 2) in response to a range of stimuli, including chemokines, cytokines, bacteria, and bacterial products at sites of inflammation. They contain membrane receptors, surface proteins, including the anti-inflammatory annexin-1 and PS, as well as active neutrophilic enzymes [78,79,80]. Although neutrophilic inflammatory EVs were reported to be generated under certain conditions [81,82], in many other studies, EVs generated by living or apoptotic neutrophils were shown to limit excessive inflammation in their surroundings. EVs are reported to orchestrate anti-inflammatory reprogramming of macrophages, reducing production of pro-inflammatory cytokines and increasing production of pro-resolving cytokines and lipid mediators, as well as their ability to efferocytose [79,83,84]. Indeed, neutrophilic EVs were shown to dampen inflammation, even in chronic inflammation, such as the rheumatic joint, acting on a range of cell types to protect cartilage [85,86]. Mechanistically, the ability of neutrophil EV to reprogramme macrophages relies on their exposure of PS and annexin-1. Annexin-1 binding to the macrophage FRP2 receptor was shown to be required for TGF-β expression. In contrast, PS exposure by EVs modulated macrophage expression of IL-1β, IL10, and IL-12. PS-binding to macrophage MerTK was moreover required to enhance the efferocytic activity of macrophages [86]. One recent study reported macrophage reprogramming by living or apoptotic neutrophils, even in the absence of efferocytosis, and identified that this involved transcriptional reprogramming of the macrophages and suppression of NF-κB activation [87]. However, it remains to be formally established whether this observation was indeed due to neutrophil EVs.

### 4.7. Reactive Oxygen Species (ROS)

Due to their essential function in microbial killing by neutrophils, ROS are vital for host immunity, but excessive ROS generation can drive oxidative damage to the host; for this reason, the generation of ROS is tightly controlled. Key to the generation of ROS is the activation of the NADPH oxidase, which occurs in a strictly regulated fashion that involves phosphoinositide-driven translocation events, as well as protein phosphorylation. The NADPH oxidase is assembled at a membrane (typically either phagosomal or plasma membrane); it is made up of membrane associated (gp91^phox^ and p22^phox^) and cytosolic components (p67^phox^, p47^phox^, p40^phox^) in the presence of activated (GTP-loaded) Rac which binds p67^phox^ (reviewed in [88]). Once assembled, the NADPH oxidase transfers NADPH-derived electrons to molecular oxygen, generating superoxide on the outside of the cell (which may be the inside of the phagosome). Further enzymatic reactions that are less tightly controlled generate increasingly more cytotoxic species, culminating in the generation of hypochlorous acid from hydrogen peroxide by myeloperoxidase, which is delivered from primary/azurophilic granules [88]. Missense mutations in any one of the subunits of the NADPH oxidase affect its function in human and mouse, causing chronic granulomatous disease (CGD) in humans [89]. Fascinatingly, ROS also have anti-inflammatory functions, at least some of which are due to their key role in apoptosis. Phagocytosis of microorganisms triggers activation of the NADPH oxidase, which in turn promotes PICD ([36,90] and Figure 1B). The same observation applies to immune complex-induced neutrophil apoptosis [40]. Experimental evidence suggests that the NADPH oxidase promotes efferocytosis of apoptotic PS-exposing neutrophils for the optimal resolution of inflammation. NADPH oxidase-dependent oxidation of the fatty acyl groups of PS culminated in the generation of lyso-PS, generating improved ligands for PS receptors, including CD36 and G2A [91,92]. Interestingly, ROS-dependent lyso-PS generated by viable neutrophils even promoted efferocytosis of these non-apoptotic cells, suggesting a potential role of lyso-PS in orchestrating the removal of excess neutrophils following their recruitment to inflammatory sites [93].

In keeping with these observations, mice lacking NADPH oxidase components were found to be prone to acute hyperinflammatory reactions in sterile inflammation models, exhibiting excessive inflammatory cell infiltrates and pro-inflammatory cytokine signatures (e.g., [94,95]). Failure of prompt efferocytosis of apoptotic cells promoted not only acute inflammation, but, in the long run, development of autoimmune disease, including SLE [96]. Genome-wide association studies identified patients carrying NADPH oxidase subunit mutations that reduced its activity in SLE cohorts (e.g., [68,97]). These observations also held true in mouse models [98,99,100]. Efferocytosis of apoptotic neutrophils by CGD macrophages, or those from CGD mouse models, was also reported to be affected: CGD macrophages were found to suffer from poor efferosome acidification and delayed clearance of ingested apoptotic cells [101]. Impaired efferocytosis resulted in poor macrophage reprogramming from pro-inflammatory (M1) to pro-resolving (M2), and reduced generation of anti-inflammatory mediators, including TGF-β and prostaglandin D2 (PGD2) [102,103,104]. In this regard, it is also interesting that, in the context of liver repair after acute acetaminophen (paracetamol) injury in the mouse, H_2_O_2_ released by neutrophils was sufficient to promote inflammatory monocyte differentiation to pro-repair macrophages by activating AMPK [17], even in the absence of neutrophil apoptosis and efferocytosis.

Taken together, these observations suggest important anti-inflammatory roles of NADPH oxidase-mediated generation of ROS, that counterintuitively suppress excessive bystander host injury from being generated due to neutrophilic inflammation.

## 5. Necrosis

Primary, or accidental, necrosis is an unregulated cell death that occurs in response to extreme exogenous stress (Figure 1). Primary necrosis is associated with cytoplasmic swelling, dilation of organelles, plasma membrane rupture, mitochondrial hyperpolarisation, oxidative burst, and release of intracellular contents into the environment. Neutrophil primary necrosis can be triggered in response to a range of situations, including some infections. Due to the release of intracellular damage-associated molecular patterns (DAMPs) into the environment, necrosis is pro-inflammatory, activating an array of mostly TLR receptors on innate immune cells. In this way, primary necrotic cells may induce NF-κB activation in viable macrophages and fibroblasts, stimulating the production of pro-inflammatory cytokines, including IL-1 and neutrophil-active chemokines [105].

Apoptotic neutrophils undergo secondary necrosis if not cleared in a timely fashion (Figure 1). Although secondary necrotic neutrophils are also characterised by a loss of plasma membrane integrity, they are less pro-inflammatory than their counterparts that have undergone primary necrosis. This is because the inflammatory activity of DAMPs released by secondary necrotic neutrophils is limited due to degradation mediated by the apoptotic machinery [106]. In some pathological situations, secondary necrotic neutrophils persist, e.g., if the ability of macrophages to efferocytose apoptotic cells is overwhelmed, or due to a genetic defect. Contrasting with primary necrotic cells, secondarily necrotic neutrophils stimulate the adaptive immune response, promoting the generation of autoantibodies, as observed in SLE.

Perhaps surprisingly, there are some reports of anti-inflammatory functions, even of necrotic neutrophils. Elastase released by neutrophils that had undergone primary necrosis activated pro-inflammatory cytokine secretion of resting macrophages. This contrasted with mixed populations of apoptotic and secondary necrotic neutrophils, which behaved similarly to early apoptotic neutrophils in terms of cytokine production elicited [107]. According to one report, even highly enriched secondary necrotic neutrophils (>95% purity) were taken up as efficiently as early apoptotic neutrophils by macrophages. This suggests that, even after undergoing secondary necrosis, these neutrophils retained some anti-inflammatory features [108]. Further studies also identified anti-inflammatory functions of apoptotic, but also primary and secondary necrotic neutrophils, on monocyte-derived human, or bone marrow derived mouse, neutrophils. Mediated by a soluble factor, the antibacterial peptide, α-defensin, released by apoptotic and necrotic neutrophils, but not other necrotic cell types, were able to inhibit the production of pro-inflammatory cytokines by macrophages in vitro, while also augmenting their antibacterial capacity in vivo [109], suggesting that even necrotic neutrophils have some anti-inflammatory functions. Follow-up studies identified the mechanism for this unexpected finding. α-defensins that had been released by dying neutrophils entered and accumulated in macrophages to inhibit bulk protein translation, but not transcription, RNA stability, or protein synthesis [110].

## 6. NETosis—Anti-Inflammatory Functions of Neutrophil Extracellular Traps

NETs are web-like structures that consist of strands of decondensed chromatin decorated with cytocidal proteins, including citrullinated histones, neutrophil proteases, myeloperoxidase, and antibacterial peptides (Figure 1). Although described as a neutrophil cell death mechanism, vital NET release by neutrophils has since also been demonstrated. NET generation is dependent upon peptidyl arginine deiminase 4 (PAD4) mediated histone citrullination, which in turn promotes chromatin decondensation. NET release is moreover dependent upon ROS and myeloperoxidase-mediated activation of neutrophil elastase, which processes histones such that chromatin packaging is disrupted [111]. NETs are released in response to a range of stimuli, including bacteria, immune complexes, uric acid crystals, phorbol esthers, and calcium ionophores. They hold an important function in host defence, trapping and killing pathogens, but they are also inflammatory and immunogenic (reviewed in [111]).

Interestingly, in addition to the direct and indirect antibacterial effects of NETs, and numerous reports on NET-dependent augmentation of inflammation, including in the context of autoimmune disease, there is also accumulating evidence for anti-inflammatory effects of NETs. Loss of function of the protease cathepsin C, as occurs in Papillon–Lefèvre syndrome, profoundly affects neutrophil ability to generate NETs, but not bacterial killing [112,113]. Interestingly Papillon–Lefèvre patients suffer from early onset periodontitis and severe skin inflammation resembling psoriasis [114]. Strikingly, mice with an impaired ability to generate NETs developed more severe disease in the pristane-model of SLE [115], suggesting that NETs may be anti-inflammatory in this context, although this finding is model-dependent with other animal models of SLE being promoted by NETs (e.g., [116,117]. NET-associated proteases were also proposed to have anti-inflammatory effects in gout, where uric acid crystals are surrounded by dense NETs that are located amongst (activated) immune cells. NET-associated proteases were shown to reduce the local concentration of proinflammatory cytokines and chemokines, many of which are highly susceptible to proteolytic degradation. In doing so, neutrophils may, via aggregated NETs, inhibit the development of excessive inflammation in certain situations [118,119]. Outside of experimental models in the mouse, aggregated NETs were shown to be active in the healthy human eye where they degrade inflammatory mediators which may accumulate during sleep at night [120]. Finally, NETs in thrombotic events, which are frequently described in pathological situations [121,122], have recently also been associated with a pro-repair function in healing the gut. Biopsies from ulcerative colitis patients suggest that mucosal erosions feature fibrin deposits that include blood clots harbouring aggregated neutrophils and NETs. Experimentally inflicted damage in the gut of mice, even in the absence of bacteria, identified an important contribution of neutrophil NET release to generating durable blood clots in lesion repair, reducing rectal bleeding. Interestingly, neutrophils involved displayed an altered transcriptional signature, which included the specific upregulation of *Padi4*, suggesting that the release of anti-inflammatory aggregated NETs in this context is an actively regulated process. Indeed, PAD4-deficient animals were characterised by diminished clot remodelling on gut lesions and delayed wound repair in a mouse model of colitis [123].

## 7. Concluding Remarks

Acute and chronic inflammation pose formidable challenges, representing the most significant cause of death in the world today. Therapeutic interventions remain limited, with few options available to date that are able to promote the resolution of inflammation. Neutrophils are generally regarded as pro-inflammatory cells that generate significant ‘bystander’ host injury. Yet, as discussed here, neutrophils also hold important anti-inflammatory functions. The best understood of these is that apoptotic neutrophils orchestrate their own efferocytosis and subsequent reprogramming of macrophages to promote resolution and tissue repair. Many aspects related to this are being explored for potential translation in the clinic. Approaches used to promote neutrophil apoptosis and macrophage reprogramming that successfully reduced inflammation in a range of in vivo models include cyclin-dependent kinase inhibitors and nanoparticles [124,125,126,127]. One recent study for example employed engineered apoptotic neutrophil bodies to enhance macrophage efferocytosis and reprogramming. Therapeutic administration of the engineered apoptotic neutrophil bodies three days after the induction of myocardial infarction in an animal model promoted the resolution of inflammation and subsequent cardiac tissue repair compared to sham controls [128].

As is evident from the above brief discussion of anti-inflammatory functions of NETs, mechanisms outside of neutrophil apoptosis exist by which neutrophils promote repair. In some cases, neutrophil cell death does not appear to be involved at all. Two recent studies reported neutrophil-mediated repair of the inflamed liver in two different liver inflammation/fibrosis models in the mouse, with two different mechanisms proposed to regulate macrophage reprogramming. In carbon-tetrachloride induced liver inflammation, neutrophils reprogrammed macrophages via microRNA miR-223, which downregulated NLRP3 inflammasome activity [16]. In contrast, in paracetamol-induced liver inflammation, reactive oxygen species (H_2_O_2_), were found to promote macrophage reprogramming [17]. At present, it remains unclear whether the validity of some of these insights may be restricted to particular disease models, or whether they may work together and/or in tandem with the more established anti-inflammatory functions involving neutrophil apoptosis.

Moreover, macrophages are clearly not the only cell type via which neutrophils exert anti-inflammatory functions. A recent report identified that neutrophilic lyso-PS triggered tissue repair in an experimental model of colitis. In this scenario, tissue repair was shown to be due to GPR34 receptor-mediated activation of type III innate lymphoid cell-dependent IL-22 production [129]. Clearly, the full functional complexity of neutrophil anti-inflammatory functions remains to be unravelled. Additional future work will be required to fully elucidate the mechanisms that underpin anti-inflammatory neutrophil functions, and successfully translate insights to the clinic.
